# Advances in the Knowledge of the Molecular Biology of Glioblastoma and Its Impact in Patient Diagnosis, Stratification, and Treatment

**DOI:** 10.1002/advs.201902971

**Published:** 2020-03-12

**Authors:** Belén Delgado‐Martín, Miguel Ángel Medina

**Affiliations:** ^1^ Department of Molecular Biology and Biochemistry Faculty of Sciences Campus de Teatinos s/n University of Málaga Málaga E‐29071 Spain; ^2^ IBIMA (Biomedical Research Institute of Málaga) Málaga E‐29071 Spain; ^3^ CIBER de Enfermedades Raras (CIBERER) Málaga E‐29071 Spain

**Keywords:** cancer therapy, diagnostics, glioblastoma, patient stratification

## Abstract

Gliomas are the most common primary brain tumors in adults. They arise in the glial tissue and primarily occur in the brain. Low‐grade tumors of World Health Organization (WHO) grade II tend to progress to high‐grade gliomas of WHO grade III and, eventually, glioblastoma of WHO grade IV, which is the most common and deadly glioma, with a median survival of 12–15 months after final diagnosis. Knowledge of the molecular biology and genetics of glioblastoma has increased significantly in the past few years, giving rise to classification methods that can help in management and stratification of glioblastoma patients. However, glioblastoma remains an incurable disease. Glioblastoma cells have acquired genetic and metabolic adaptations in order to sustain tumor growth and progression, including changes in energetic metabolism, invasive capacity, migration, and angiogenesis, that make it very difficult to find suitable therapeutic targets and to develop effective drugs. The current standard of care for glioblastoma patients is surgery followed by radiotherapy plus concomitant and adjuvant chemotherapy with temozolomide. Although progress in glioblastoma therapies in recent years has been more limited than in other tumors, numerous drugs and targets are being proposed and many clinical trials are underway to develop effective subtype‐specific treatments.

## Graphical abstract

Glioblastoma occurs in the brain and is the most common and deadly glioma, with a median survival of 12 to 15 months after final diagnosis. It remains as an incurable disease. Different aspects of the molecular biology and clinical management of glioblastoma, with a special focus on new therapeutic strategies under development, are summarized.

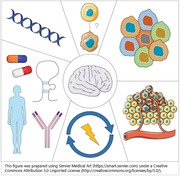

## 1 Introduction

Gliomas are the most common malignant brain tumors in adults. They can arise anywhere in the central nervous system (CNS), but they occur mainly in the brain, from glial tissue. Its incidence rate is from 4.67 to 7.73 per 100 000 people. The World Health Organization (WHO) classifies gliomas in grades ranging from I to IV according to their aggressiveness, with glioblastoma (GBM) being a grade IV glioma, meaning that it is the most aggressive of them all, with an incidence of 0.59 to 3.69 per 100 000 people.^[^ ^1^ ^]^ This incidence increases with age, reaching a maximum between 75 and 84 years, and is higher in white males. GBM represents 12–15% of all intracranial tumors and 50–60% of astrocytic tumors and presents a poor prognosis, since less than 10% of patients survive more than 5 years, with an average survival of 12–15 months after the final diagnosis.^[^ ^2^ ^]^

From the first microscopic observations to the latest studies of gene expression, various classifications have been made in order to achieve a better understanding of the disease. Traditionally, GBMs have been separated into primary and secondary GBMs. Hans‐Joachim Scherer, a German neuropathologist, was the first to introduce the distinction between primary and secondary GBMs.^[^ ^3^ ^]^ Although there were recognizable clinical differences between these two subtypes, it was not clear until 1996 that they were histopathologically distinguishable too.^[^ ^4^ ^]^ Now we know that these two major subtypes carry distinct genetic alterations, and this is used as a criterion for patient stratification and prognosis.

The vast majority of GBMs (≈90%) arise de novo in elderly patients, without clinical or histological evidence of a less malignant precursor lesion; they are the so‐called primary GBMs. Secondary GBMs arise from lower grade gliomas: diffuse astrocytoma or anaplastic astrocytoma. They appear in younger patients, have a lower degree of necrosis, are usually located in the frontal lobe and their prognosis is significantly better than that of primary GBMs. Histologically, primary and secondary GBMs are practically indistinguishable, but they differ in their genetic and epigenetic profiles,^[^ ^5^ ^]^ as will be detailed later.

Subsequently, large‐scale genomic studies began to analyze tumor heterogeneity and to identify tumor subtypes that would allow the stratification of patients for their treatment. In 2010, the cancer genome atlas (TCGA) took a big step in this direction, identifying four clinical subtypes of GBMs according to their expression profile in a study with 200 GBM samples.^[^ ^6^ ^]^ The classification previously established by the WHO in 2007 was based on histopathological characteristics that often did not correspond to the clinical characteristics of the tumor and did not allow an adequate stratification of patients.^[^ ^7^ ^]^ Therefore, this new classification established by Verhaak et al. represented a significant advance in both the molecular knowledge of the disease and the clinical field, since there is a correlation between the defined subtypes and the clinical manifestations of the tumor.^[^ ^6^ ^]^ In 2016, the WHO carried out an update of the classification of CNS tumors based on molecular criteria,^[^ ^8^ ^]^ leaving behind the already obsolete histological criterion established in the 2007 classification.

GBMs lead to a rapid clinical deterioration, with a median survival prognosis of 15 months, even using very aggressive therapies. The current treatment is based on surgery for resection followed by chemotherapeutic agent temozolomide (TMZ) plus radiotherapy,^[^ ^9^ ^]^ but GBM is still considered an incurable disease. In order to develop new therapeutic strategies, it is critical not just to know the pathways and mutations involved in GBM, but to reach a global understanding of the disease for the search of effective therapeutic targets. GBM was the first tumor characterized by TCGA, whose molecular studies identified three important genetic events in human GBM: 1) dysregulation of growth signaling via amplification and mutational activation of receptor tyrosine kinase (RTK) genes, 2) activation of the phosphatidylinositol‐3‐OH‐kinase (PI(3)K) pathway, and 3) inactivation of the p53 and retinoblastoma tumor suppressor pathways.^[^ ^10^ ^]^

Even though many details regarding GBM remain unknown, the discovery of some genetic and molecular features has led to a better comprehension of the disease, which allows for a more accurate stratification of patients and the identification of new possible targets. These findings will be reviewed in the present work.

## 2 Histopathologic and Molecular Classification of Gliomas

There are many ways to classify gliomas, but the most general classification is based on the degree of invasiveness of the adjacent tissue. According to this, GBM can be separated in two major categories: 1) gliomas with diffusive growth that showcase the ability to infiltrate surrounding brain parenchyma and often recur after total resection, and 2) gliomas with circumscribed growth that can be cured by resection alone. Low‐grade diffuse gliomas of WHO grade II tend to progress to high‐grade gliomas of WHO grade III and eventually WHO grade IV, that is, GBM.^[^ ^11^ ^]^ This being said, according to their malignancy, gliomas have been classified in four grades by WHO: WHO grade I gliomas include tumors with low proliferative potential; WHO grade II gliomas are those with infiltrative capacity and recurrence, but they show low proliferative activity; WHO grade III gliomas show histological evidence of malignancy; WHO grade IV gliomas include tumors that, apart from the features of the latter, showcase necrosis and microvascular proliferation, like GBM does.^[^ ^8^, ^12^ ^]^

Gliomas arise in the glial tissue and can be either astrocytic, oligodendrocytic or oligoastrocytic. GBM can be divided into different subgroups and each one of them is associated with a different signature. In 2016, the WHO carried out an update of the classification of the brain tumors established in 2007, discarding the obsolete principle of diagnosis based on microscopy and incorporating new molecular parameters to define tumor identities. This new classification includes GBM in the diffuse astrocytic and oligodendroglial tumors group and divides it into three subgroups based on isocitrate dehydrogenase (IDH) mutations: 1) glioblastoma, IDH‐wildtype, clinically identified as primary GBM or de novo GBM and predominant in patients over 55 years of age (10% of cases), 2) glioblastoma, IDH‐mutant, clinically identified as secondary GBM and more common in younger patients (90% of cases), and 3) glioblastoma NOS (not otherwise specified), which does not fit into the other categories and is not well defined (**Table** **1**). Furthermore, glioblastoma, IDH‐wildtype can be divided into different subtypes based on histologic features: 1) gliosarcoma, with a metaplastic mesenchymal component; 2) giant cell glioblastoma, characterized by the presence of multinucleated cells; and 3) epithelioid glioblastoma, a provisional new variant of GBM added to the classification in 2016, which features large epithelioid cells and variably present rhabdoid cells.^[^ ^8^ ^]^

**Table 1:** Comparison of molecular subtypes established by WHO in 2016

	*IDH*‐wildtype glioblastoma	*IDH*‐mutant glioblastoma
Synonym	Primary glioblastoma	Secondary GBM
Mean age at diagnosis	56–61	32–48
Proportion of cases	10%	90%
Precursor lesion	Nonexistent; develops de novo	Diffuse astrocytoma Anaplastic astrocytoma
Median overall survival Surgery + radio/chemotherapy	15 months	31 months
Location	Supratentorial	Preferentially frontal
Necrosis	90%	50%
Main genetic aberrations	*TERT* promoter mutations, *EGFR* amplification, deletion of *PTEN*	*IDH*, *TP53*, *ATRX* mutations; methylation of *MGMT* promoter
Transcriptional profiles^a)^	Neural, proneural, mesenchymal, classical	Proneural

a) According to ref. [6] and data from ref. [5].

## 3 Classification of Glioblastomas Based on Their Genetic Expression Profiles

The molecular patterns of GBM can partially explain clinical outcomes and predict responses to treatment. Classification methods are important for the development of targeted therapies for individual subtypes, since GBM is a complex and heterogeneous disease. Molecular classification of GBM has evolved over the years in order to achieve a better comprehension of the molecular events that drive oncogenesis and progression.^[^ ^13^ ^]^ Gene expression profiling of GBM allowed the identification of several molecular subgroups.

In 2006, Philips et al. identified three molecular subtypes of high‐grade astrocytoma with significant prognostic value that were named proneural, proliferative and mesenchymal, according to the genes that characterize each group. Proliferative subtype exhibited overexpression of markers of proliferation compared to the other subtypes. Mesenchymal tumors displayed overexpression of markers of angiogenesis. Proneural tumors expressed genes associated to normal brain and the process of neurogenesis and were associated with better survival than the other two subclasses. These results were later used to classify GBM samples, resulting in a subtype classification with prognostic value.^[^ ^14^ ^]^

Later, another molecular classification was established using an unsupervised hierarchical clustering analysis. The classification of GBMs established by the WHO in 2007 was based on histological features that did not allow a proper stratification of patients,^[^ ^7^ ^]^ so Verhaak et al., in 2010, carried out a study of the genetic expression profiles of 200 GBM samples in order to provide a new and more precise form of classification, based on molecular features. By the integration and analysis of multi‐dimensional genomic data, they identified four clinically relevant subtypes of GBM characterized by abnormalities in *PDGFRA*, *IDH1*, *EGFR*, and *NF1*. These are proneural, neural, classical and mesenchymal subtypes. Proneural class is enriched in oligodendrocytic signature and is the one with better prognosis. Neural GBMs show association with oligodendrocytic and astrocytic signature but are also enriched in neuron‐related genes. It is the worst defined subtype, since the genetic expression profile is similar to normal brain tissue. Classical group is strongly associated with murine astrocytic signature. Mesenchymal class exhibits mesenchymal phenotype and expresses Schwann cell markers and microglial markers.^[^ ^6^ ^]^

## 4 Primary Glioblastoma versus Secondary Glioblastoma

Histologically, primary and secondary GBMs are almost identical, but they have different genetic and epigenetic profiles. Primary GBMs harbor three main genetic aberrations, which has been confirmed by the analysis of single nucleotide polymorphisms (SNPs): 1) amplification and/or high rate of *EGFR* mutation in chromosome 7p, 2) homozygous deletion of *CDKN2A‐p16^INK4a^* in chromosome 9p, and 3) deletion of *PTEN*, frequently associated with monosomy 10.^[^ ^15^ ^]^ Amplification of oncogene *MDM2* has also been observed, specially, in tumors with no *TP53* and *TERT* mutations. Other genetic aberrations were described in the TCGA study of GBM in 2008, such as *NF1* mutations and homozygous deletion of *PI3KR1*.^[^ ^10^ ^]^

In contrast to primary GBMs, *TP53* mutations, associated with methylation of the promoter of *MGMT*, are observed in most secondary GBMs, along with partial loss of heterozygosity of 10q, 13q, 19q, and 22q.^[^ ^16^ ^]^ However, the identification of *IDH1* as a molecular marker was crucial for the separation of these two subtypes. They were first identified by Yan et al. in 2009, when they found out that these mutations occurred in most patients with secondary GBM and were associated with an increase in overall survival OS).^[^ ^17^ ^]^ Nowadays, after subsequent studies regarding this issue, it is agreed that *IDH1* mutation is the most reliable diagnostic molecular marker of secondary GBMs.^[^ ^5^ ^]^

## 5 Adult Glioblastoma versus Pediatric Glioblastoma

High‐grade gliomas comprise 15–20% of CNS tumors in children and 70–90% of patients die two years after diagnosis. Adult GBMs and pediatric GBMs differ in frequency, anatomic location and pathology, suggesting that progenitor cells, mature cells and tumor microenvironment (TME) affect the disease process. Pediatric GBMs arise in brain regions in which adult GBMs rarely occur and they usually develop de novo, which means they are primary GBMs. Because of this, *IDH1* mutations are seldom observed in these tumors.^[^ ^18^ ^]^

By analyzing the expression profile in a cohort of GBMs from children and adult patients, Sturm et al. found that pediatric GBMs exhibit mutations in *H3F3A* and *DAXX*, rarely seen in adult GBMs.^[^ ^19^ ^]^ These genes, which encode proteins involved in chromatin remodeling, allowed Sturm et al. to establish six distinct DNA methylation clusters: 1) “IDH,” 2) “K27,” 3) “G34,” 4) “RTK I (PDGFRA),” 5) “mesenchymal,” and 6) “RTK II (classic).” When comparing these subtypes with those established by Verhaak et al.,^[^ ^6^ ^]^ it is observed that there are similarities between the RTK II (classic) subtype and the classical subtype, and between the RTK I (PDGFRA), IDH and K27 subtypes, and the proneural subtype.^[^ ^19^ ^]^

## 6 Cellular Origin and Tumor Heterogeneity

The low frequency of GBMs and other brain tumors compared to other types of tumor may indicate that there is a high degree of protection of the brain against genotoxic stress. ATP‐binding cassette transporters located in the blood–brain barrier (BBB) seem to have an important role in this protection, since they restrict diffusion of mutagenic agents to the brain. DNA is especially sensitive to these agents during replication, but most brain cells are in a post‐mitotic state.^[^ ^20^ ^]^

Neural stem cells (NSC) and glial progenitor cells have been found in many regions of the adult brain. These populations, referred to as neural stem and progenitor cells (NSPC), are located in the subventricular zone, the subcortical white matter and the dentate gyrus of the hippocampus. These NSPC, in addition to differentiated adult glia, may constitute a subpopulation called brain tumor initiating cells or stem‐like cells or glioma stem cells (GSC), as referred to here. They show some features commonly associated with cancer, like self‐renewing capacity, robust proliferative potential and multilineage differentiation.^[^ ^21^ ^]^ In 2003, Singh et al. identified cancer stem cells (CSCs) in brain tumors by expression of the cell surface marker CD133. These cells showed capacity for proliferation, self‐renewal and differentiation, lacked the expression of neural differentiation markers and was necessary for the proliferation and self‐renewal of the tumor in culture.^[^ ^22^ ^]^ Since then, many studies have been carried out to determine if these CD133‐positive cells correlate with worse prognosis.^[^ ^23^ ^]^

The relationship between the GSC and the four clinical subtypes established by Verhaak et al. remains unclear, but expression profiling of GSC from primary GBMs makes it possible to divide them into two groups: type I (proneural signature) cells, similar to fetal NSC, CD133‐positive and CD15‐positive; and type II (mesenchymal signature), similar to adult NSC, CD133‐negative and CD44‐positive, more invasive and with semi‐adherent growth (**Table** **2**).^[^ ^24^ ^]^ CD133‐positive cells can undergo asymmetric division to form CD133‐negative daughter cells (note that these daughter cells show different properties from CD133‐negative cells) and are more tumorigenic based on traditional criteria,^[^ ^25^ ^]^ although CD133‐negative cells can form tumors and produce CD133‐positive progeny in vivo.^[^ ^26^ ^]^ It has been observed that proneural subpopulations of GBM undergo a shift to mesenchymal subtype in response to radiation, enhancing resistance to radiation and increasing the invasiveness of the cells. This could mean that CD133‐negative GSCs, which represent mesenchymal subtype, might be even more resistant to radiotherapy and chemotherapy than CD133‐positive GSCs,^[^ ^27^ ^]^ which have been proved to be resistant to conventional anticancer therapies.^[^ ^28^ ^]^ All these and other findings related to CD133 suggest that its use as a marker may not be accurate, since its expression varies strongly depending on diverse factors, and there could be other markers that explain the switch in cell behavior in a more proper way, like snail.^[^ ^29^ ^]^

**Table 2:** Comparison of type I and type II GSCs

	Type I	Type II
CD133 marker	+	−
Transcriptional profile^a)^	Proneural	Mesenchymal
Molecular signature	Fetal NSC	Adult NSC
Growth	Gliomaspheres	Semi‐adherent, invasive
Tumorigenic potential	High	Low

a) According to ref. [6] and data from ref. [5].

The TCGA study and other massive analysis have shown that GBM is a very heterogeneous tumor and that is why it is possible to establish a classification based on molecular pathogenesis and driver mutations. Despite all these studies, the combination of the omics results obtained still cannot explain the complex cellular processes that take place in the tumor mass and tumor heterogeneity.

**Figure 1:**
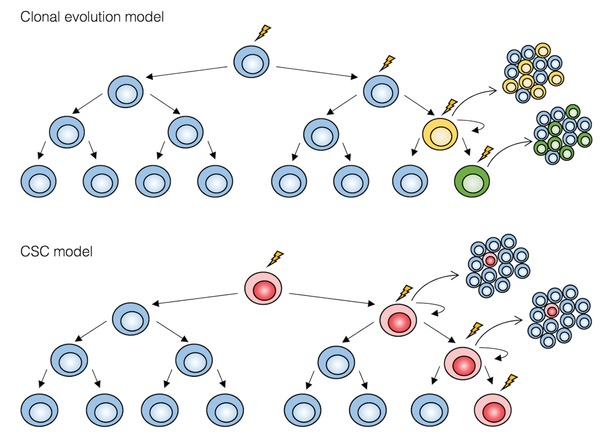
Two models for tumor heterogeneity. In the clonal evolution model, all undifferentiated cells have similar tumorigenic capacity. In the CSC model, only CSCs (in red) can sustain tumor growth, thanks to their self‐renewal properties and enormous proliferative potential. Oncogenic events are represented by a thunderbolt.

Two models have been proposed to explain tumor heterogeneity (**Figure** **1**). Clonal evolution model suggests that genetic and epigenetic changes occur in single cells and if such changes confer a selective advantage, these cells out‐compete other clones. As the tumor progresses, the genomic instability increases, and new genetic variants appear. The presence of these genetic variants explains the tumor heterogeneity. This model is supported by the existence of common mutations in G1/S cell cycle checkpoint, RTK/MAPK/PI3K and *TP53*.^[^ ^10^, ^30^ ^]^ CSC model proposes a hierarchical organization of cells within the tumor, in which only CSCs have the ability to sustain tumor growth and give raise to phenotypically diverse cancer cells. These two models are not mutually exclusive, as CSCs themselves undergo clonal evolution and acquire more aggressive self‐renewal or growth properties.^[^ ^31^ ^]^

In the brain parenchyma, GSCs are located in specific areas and reside in the presence of different components and other types of cells. This is called the TME, a complex dynamic entity in which a bidirectional communication is established between GSCs and all those elements. The niche can affect the biology of GSCs, but there is also evidence that GSCs can modulate the TME in order to satisfy their requirements.^[^ ^32^ ^]^ Evidence supporting the CSC model in GBM has been obtained by comparison of transcriptomic profiles showing that human GBMs harbor transcriptomes similar to purified mouse astrocytes, neurons, oligodendrocyte progenitor cells (OPC), and NSCs.^[^ ^6^ ^]^ This model suggests that intrinsic and extrinsic biological properties of the cell of origin may strongly influence tumor pathogenesis, which may be particularly important for tumors originating from mature astrocytes, the most common and heterogeneous cells in the mammalian brain. Indeed, intrinsic astrocyte heterogeneity has been demonstrated to influence tumor growth and malignant progression in glioma mouse models. It remains unclear if this affects glioma pathogenesis in the adult mammalian brain.^[^ ^33^ ^]^

A great source of heterogeneity in TME is the abundance of parenchymal cells, such as vascular cells, microglia, peripheral immune cells, and neural precursor cells.^[^ ^34^ ^]^ Up to 30% of all cells in glioma biopsies are recognized by macrophage markers.^[^ ^35^ ^]^ In 2000, Badie and Schartner carried out some experiments to determine whether glioma‐associated microglia could be recruited de novo from the brain microglial population or migrate toward brain tumors from the periphery.^[^ ^36^ ^]^ They found that macrophages are primarily detected within brain tumors, while microglia are present in all brain tissue. A study carried out by Chen et al. in 2017 agrees that bone‐marrow‐derived monocyte/macrophages predominate within the GBM parenchyma, while microglia reside at the tumor periphery.^[^ ^37^ ^]^ According to this study, infiltrating macrophages represent ≈85% of the total TAM population and microglia accounts for the remaining 15%. The infiltration of macrophages occurs because of the impairment of the blood–brain barrier, which is a typical condition in neuropathologic diseases. Many studies have shown that tumor‐associated macrophages (TAMs) contribute to tumor progression by creating a supportive stroma for tumor cells expansion and invasion. TAMs are recruited to the glioma and produce cytokines and other factors that promote glioma cells proliferation and migration.^[^ ^38^ ^]^ Recently, Martinez‐Lage et al. carried out a comprehensive immunohistochemical study to characterize the immune landscape associated with the four GBM molecular subtypes defined by Verhaak et al.^[^ ^39^ ^]^ They found that the most immunogenic subtype was the mesenchymal subtype, followed by neural, classical, and proneural GBMs. They also determined that medium and high levels of CD163^+^ macrophage lineage is correlated to patients' survival, with medium levels of infiltration having a worse impact in survival than high levels.

TME is particularly intricate in gliomas. Glioma cells are surrounded by neurons, which create a “electrochemical microenvironment” that affects tumor growth. In 2015, Oswald et al. demonstrated the presence of large membrane protrusions called microtubes in astrocytomas that allow tumor cells to form an interconnected network. This network is able to communicate via connexin 43 (Cx43) gap junctions and could be used as a route for brain invasion, proliferation and interconnection over long distances.^[^ ^40^ ^]^ In the same year, Venkatesh et al. showed that neuronal activity promotes high‐grade glioma proliferation and growth through neuroligin‐3 (NLGN3) secretion. NLGN3 acts as a mitogen by recruiting PI3K‐mTOR oncogenic pathway to induce glioma cells proliferation.^[^ ^41^ ^]^ In 2019, two studies showing the electrical and synaptic integration of glioma cells in neuronal circuits were published.^[^ ^42^, ^43^ ^]^ Both studies demonstrated the existence of spontaneous, excitatory postsynaptic potentials in tumor cells mediated by α‐amino‐3‐hydroxy‐5‐methyl‐4‐isoxazolepropionic acid receptors, which could bring to the fore new therapeutic strategies for high‐grade gliomas.

While cell lineage is unidirectional and hierarchical in the normal brain, cancer cell lineage is plastic, and the differentiation state of the tumor cells is dynamic, as well as the interactions between tumor cells and their microenvironment. Any classification system is based on a snapshot view of the tumor and it is not possible to make predictions of how a concrete tumor will evolve over time based on them, as some tumors are able to shift from one subtype to another. It has also been shown that the mutations present in recurrent GBM are not fully represented in primary GBM and chemotherapeutic agents are responsible for the selection of malignant clones.^[^ ^44^ ^]^

## 7 Events Involved in Gliomagenesis

The vast majority of GBMs develop de novo (90%), while secondary GBMs are uncommon (10%). They progress through the acquisition of different molecular alterations, *IDH* mutations being the most decisive ones. *IDH* mutations are common in secondary GBM (85%) and rarely found in primary GBM (5%). They drive the tumor progression in early phases and are positively correlated with other genetic abnormalities found in low‐grade gliomas, like *TP53* and *ATRX* mutations and 1p/19q co‐deletion; they display an inverse correlation with *EGFR* gene amplification and monosomy of chromosome 10, which are common events in primary GBM.^[^ ^17^ ^]^ In spite of these differences, the TCGA study identified, in 2008, three important genetic events in human GBMs: 1) dysregulation of growth signaling via amplification and mutational activation of RTK genes, 2) activation of the phosphatidylinositol‐3‐OH‐kinase (PI(3)K) pathway, and 3) inactivation of the p53 and retinoblastoma tumor suppressor pathways.^[^ ^10^ ^]^ These events drive GBM malignant transformation, but tumors need a great amount of genetic and metabolic adaptations in order to maintain proliferation and expansion, including changes in energetic metabolism, invasive capacity, migration and angiogenesis.

### 7.1 Energetic Metabolism of GBM Cells

Metabolism of cancer cells differs from normal cells in its divergent utilization of available nutrients and the energetic imbalance between the cell and its mitochondria.^[^ ^45^, ^46^ ^]^ For cancer cells, nutrient availability is not as important as the ability to metabolize available nutrients into useful compounds for their growth and proliferation under stress conditions, in a process regulated by oncogenic signaling. Cancer cells are able to reprogram their metabolism in order to acquire survival adaptive advantages via genetic or epigenetic alterations of metabolism‐related genes; for example, oncogene *IDH*.^[^ ^47^ ^]^

**Figure 2:**
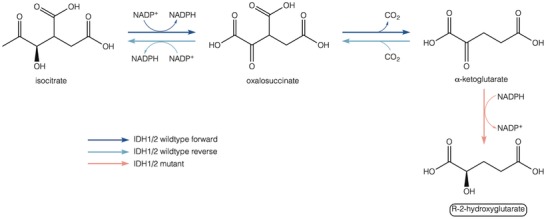
Enzyme activity of IDH1/2 wildtype and IDH1/2 mutant. IDH1/2 catalyzes the oxidative decarboxylation of isocitrate to produce α‐KG, using NADP^+^ as cofactor and producing NADPH and CO_2_ in the forward reaction. IDH1/2 mutations confer a gain‐of‐function activity that catalyzes the conversion of α‐KG into the oncometabolite R2HG in a NADPH‐dependent manner.

**Figure 3:**
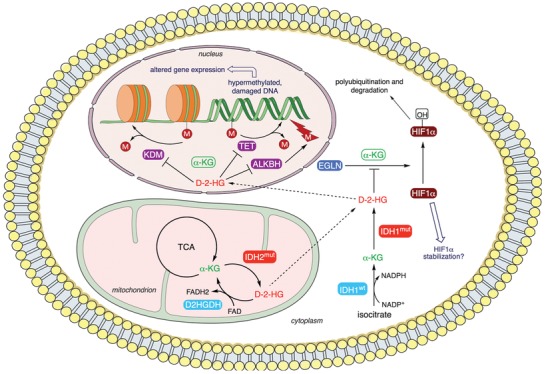
Metabolism and targets of oncometabolite R2HG. R2HG binds competitively to enzymes that normally use α‐KG as a cofactor, causing a decrease in the activity of these enzymes, including DNA demethylases (ten‐eleven translocation family of DNA methylcytosine dioxygenases; TET), histone lysine demethylases (KDM), DNA repair proteins (α‐KG/Fe(II)‐dependent dioxygenases; ALKBH) and HIF1α prolyl hydroxylases (egg‐laying deficiency protein nine‐like; EGLN). This leads to a hypermethylated genotype, which results in altered gene expression, and changes in the expression of HIF1α‐dependent genes through HIF1α stabilization. This figure was prepared using Servier Medical Art (https://smart.servier.com) under a Creative Commons Attribution 3.0 Unported License (https://creativecommons.org/licenses/by/3.0/).

IDH is an enzyme with five isoforms that catalyzes the oxidative carboxylation of threo‐*D5* isocitrate to alpha‐ketoglutarate (α‐KG) and carbon dioxide (CO_2_) (**Figure** **2**). IDH3 is located in the mitochondria and catalyzes the third step in the citric acid cycle, reducing NAD^+^ to NADH in the process; IDH1 and IDH2 catalyze the same reaction, but they use NADP^+^ instead of NAD^+^ and they do not participate in the citric acid cycle. IDH1 and IDH2 play an important role in many cellular metabolic functions, including glucose sensing, glutamine metabolism, lipogenesis, and regulation of cellular redox status; but most important, IDH maintains levels of reduced glutathione (GSH) and peroxiredoxin by providing NADPH.^[^ ^48^ ^]^ Mutations in *IDH1* and *IDH2* cause structural changes that result in the loss of affinity for isocitrate and acquisition of the ability to catalyze the NADPH‐dependent reduction of α‐KG to *R*(‐)‐*2‐*hidroxyglutarate (2HG).^[^ ^49^ ^]^ 2HG is rapidly degraded under normal physiologic conditions by D2HG dehydrogenase, but the generation of 2HG in *IDH*‐mutated cells overcome its removal in ≈1000‐fold, resulting in its accumulation. 2HG has been shown to be an oncometabolite (**Figure** **3**), since it can prevent histone demethylation, altering gene expression and inhibiting differentiation.^[^ ^50^ ^]^ Furthermore, 2HG stimulates prolyl hydroxylases, egg‐laying deficiency protein nine‐like (EGLN), involved in the hydroxylation of hypoxia‐inducible factor 1 (HIF‐1), causing its ubiquitination and degradation. HIF‐1 is a transcription factor that regulates expression of many hypoxia‐related genes and modulates angiogenesis and vascular hyperpermeability.^[^ ^51^ ^]^ Thus, degradation of 2HG occurs in tumors that exhibit *IDH* mutations, which is consistent with the idea that these tumors lack necrosis and microvascular proliferation.^[^ ^52^ ^]^

### 7.2 Invasion and Angiogenesis

Invasion of adjacent tissues and angiogenesis, described by Hanahan and Weinberg as “hallmarks of cancer,”^[^ ^53^ ^]^ are two critic events for the progression of GBM.

#### 7.2.1 The Invasion Process

**Figure 4:**
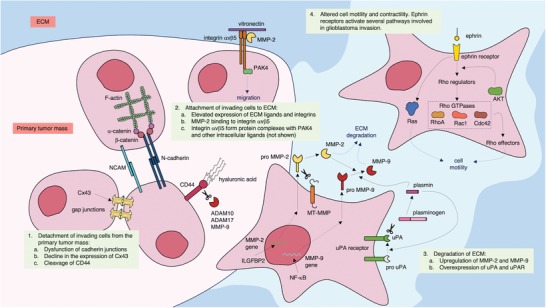
Events involved in glioma invasion. Cx43: connexin 43; NCAM: neural cell adhesion molecule; ADAM: a disintegrin and metalloproteinase; MMP: matrix metalloproteinase; PAK4: p21 activated kinase 4; ILGFBP2: insulin‐like growth factor binding protein‐2; MT‐MMP: membrane‐type matrix metalloproteinase; uPA: urokinase plasminogen activator; AKT: protein kinase B; Rac1: Ras‐related C3 botulinum toxin substrate 1; Cdc42: cell division cycle 42. This figure was prepared using Servier Medical Art (https://smart.servier.com) under a Creative Commons Attribution 3.0 Unported License (https://creativecommons.org/licenses/by/3.0/).

The process of GBM invasion involves four steps: 1) detachment of invading cells from the primary tumor mass, 2) sequential adhesion to the extracellular matrix (ECM), 3) degradation of ECM, and 4) altered cell motility and contractility (**Figure** **4**).^[^ ^54^ ^]^ Unlike other tumors, spreading of GBM outside the brain is uncommon, due to the composition of the ECM in CNS. The brain parenchyma harbors a special ECM structure, the perineuronal network, containing high levels of hyaluronan sulfate proteoglycans, chondroitin sulfates proteoglycans, tenascins, and link proteins.^[^ ^55^ ^]^ The adaptation of GBM cells to this environment enables them to invade the brain via different invasion routes: leptomeningeal space, brain parenchyma, white matter tracts of corpus callosum, and perivascular space.^[^ ^56^ ^]^

##### Detachment of Invading Cells from the Primary Tumor Mass

When tumors invade adjacent tissues, invading cells shed from the primary tumor following several steps. The first one is the dysfunction of cadherin junctions that hold the primary mass together, which has been demonstrated to be a major contributor to cancer progression.^[^ ^57^ ^]^ Cadherins form adherent junctions between adjacent cells and interact with proteins that link the receptor to fundamental intracellular processes, including arrangement of the cytoskeleton, cell signaling and vesicles traffic. Cadherins affect diverse aspects of tissue architecture, since they maintain cell‐to‐cell cohesion and contribute to morphological differentiation and contact inhibition of growth and motility.^[^ ^58^ ^]^ Thus, cadherins may function as suppressors of tumor growth and invasion.^[^ ^59^ ^]^

The second step is the decline in the expression of Cx4, which is the most abundant gap junction protein in the CNS and is expressed primarily by astrocytes.^[^ ^60^ ^]^ This leads to a reduction in the formation of gap junctions, which is correlated with increased in vivo motility of glioma cells.^[^ ^61^ ^]^ Cell growth and phenotype are partially controlled by the cell‐to‐cell exchange of growth regulatory factors through gap junctions, so the impairment of this exchange can lead to unregulated growth and neoplasia.^[^ ^62^ ^]^ Taken together, these data suggest that the decrease in the expression of Cx43 plays an important role for increased growth and invasion of gliomas.^[^ ^59^ ^]^

The third step is cleavage of CD44, which constitutes the anchor between the primary tumor mass and the ECM. CD44 is a ubiquitously expressed transmembrane glycoprotein that is involved in cell activation, cell‐to‐cell adhesion and cell‐substrate interaction.^[^ ^63^ ^]^ CD44 is cleaved by both ADAM 10 and 17 proteases and MMP‐9 in a process that promotes motility via cytoskeletal reorganization.^[^ ^64^, ^65^, ^66^ ^]^ The proteolytically released CD44 extracellular domain (CD44‐ECD) promotes glioma migration and invasion,^[^ ^65^ ^]^ while the intracellular domain (CD44‐ICD) translocates to the cell nucleus, where it acts as a transcription factor. CD44‐ICD binds to a DNA consensus sequence in the promoter region of the *MMP‐9* gene and upregulates its expression. Furthermore, HIF1α‐responsive genes respond to CD44‐ICD induction under normoxic conditions independently of HIF1α expression. Additionally, three enzyme‐encoding genes in oxidative glycolysis were found to be upregulated by CD44‐ICD too: *ALDOC*, which encodes fructose‐biphosphate aldolase c; *PDK1*, which encodes pyruvate dehydrogenase kinase‐1; and *PFKFB4*, which encodes 6‐phosphofructose‐2‐kinase/fructose‐2,6‐biphosphatase 4. These data suggest that CD44 may promote the Warburg effect (aerobic glycolysis) in cancer cells that are CD44+.^[^ ^67^ ^]^ CD44 and RHAMM, both receptors for hyaluronan, are expressed in GBM in higher levels than low‐grade gliomas or non‐neoplastic specimens of human brain.^[^ ^68^ ^]^ RHAMM expression is higher at the invasive edges of gliomas, while core regions express more CD44.^[^ ^69^ ^]^ These hyaluronan receptors are both suppressed by p53,^[^ ^70^, ^71^ ^]^ suggesting that early progression through cell cycle checkpoints and capacity of migration are related.^[^ ^72^ ^]^

##### Adhesion of Invading Cells to ECM

Interactions of the glioma cells with the ECM are mediated by integrins, which are a family of transmembrane glycoproteins adhesion receptors composed of two subunits (α and β) that set a bidirectional relationship between the ECM and intracellular signaling networks.^[^ ^73^ ^]^ Integrins have key roles in regulating cellular physiology, including aspects as polarity, proliferation, differentiation, survival, and migration.^[^ ^74^ ^]^ Consequently, failures regarding these proteins, and thus the intracellular signaling networks they control, have several pathologic implications.^[^ ^75^ ^]^ Elevated expression of ECM molecules and their integrin receptors has been found in GBM tumor samples, suggesting its involvement in GBM progression.^[^ ^76^ ^]^ Integrins αvβ3 and αvβ5 were first identified as attractive therapeutic targets in GBM, due to their high expression compared to normal brain tissue.^[^ ^77^ ^]^ Their ECM ligands, fibronectin and vitronectin, were also found to be upregulated in GBM.^[^ ^77^, ^78^ ^]^ In addition, it has also been observed that αvβ3 expression is higher at the periphery of high‐grade gliomas, while αvβ5 is expressed predominantly at the center of the tumor mass.^[^ ^79^, ^80^ ^]^ Moreover, αvβ3 colocalizes with MMP‐2 at the invasion front, supporting the role of these enzymes for invasive cell behavior. MMP‐2 and αvβ3 bind directly to each other, an interaction that depends on the C‐terminus of MMP‐2,^[^ ^81^ ^]^ and the integrin form protein complexes with p21 protein‐activated kinase 4 (PAK4), mediating invasion through regulation of migration processes and anoikis resistance.^[^ ^82^ ^]^ Recent experimental results suggest that there is firm evidence of the involvement of Ephrin receptors, Rho GTPases and casein kinase 2 (CK2) on an invasion route that could provide new therapeutic targets.^[^ ^56^ ^]^

##### Degradation of ECM

MMPs are the main responsible for the degradation of the ECM during glioma invasion. There is evidence that a direct association exists between increased expression of MMPs and tumor invasiveness, angiogenesis, development of metastases, and decreased survival time.^[^ ^83^ ^]^ The levels of MMP‐2 and MMP‐9 are specially related to tumor progression in human gliomas,^[^ ^84^ ^]^ with MMP‐2 being primarily involved in both invasion and angiogenesis and MMP‐9 contributing mostly to tumor neovascularization, due to their cellular origin and localization.^[^ ^85^ ^]^ Several factors are known to upregulate MMP expression. Low‐density lipoprotein receptor‐related protein 1 induces MMP‐2 and MMP‐9 expression via an ERK‐dependent promigratory process.^[^ ^86^ ^]^ MMP‐9 is known to be upregulated by NF‐κB,^[^ ^87^ ^]^ while MMP‐2 is known to be upregulated by insulin‐like growth factor binding protein 2 and by the Forkhead box transcription factor FoxM1B.^[^ ^88^, ^89^ ^]^ The latter not only upregulates MMP‐2 but is capable of transforming immortalized human astrocytes into invasive GBM cells via degradation of PTEN and activation of AKT.^[^ ^90^ ^]^

Urokinase plasminogen activator (uPA) is a serine protease that catalyzes the conversion of inactive plasminogen into plasmin, an enzyme that is able to degrade several ECM proteins and activate MMPs, growth factors and pro‐uPA. uPA and its receptor (uPAR) have been shown to be overexpressed in GBM.^[^ ^91^ ^]^ Binding of uPA and uPAR directs plasmin activity to the migrating tumor cell surface and results in increased tumor cell migration and invasion.^[^ ^83^ ^]^ The PI3K/AKT signaling pathway is inhibited when uPA is downregulated, suggesting that this pathway regulates uPA‐induced cell migration.^[^ ^92^ ^]^

##### Altered Cell Motility and Contractility

Cell motility requires the formation of cytoplasmic contractile force. Glioma cells migrate like nontransformed neural progenitor cells, extending a leading lamellipodium followed by forward movement of the nucleus and cell body that requires myosin II. Glioma cells specifically require A and B isoforms of myosin II in order to squeeze through pores smaller than its nuclear diameter, which is an important adaptation needed to move within the brain matrix, due to the presence of narrow extracellular spaces.^[^ ^93^ ^]^ Cell migration is a multistep process initiated by extracellular stimuli that are transduced into intracellular biochemical signals that lead to small GTPases activation and cytoskeletal reorganization. Rho‐family GTPases are molecular “switches” within cells, which control actin cytoskeletal structures and provide the molecular framework that supports directed cell motility.^[^ ^94^ ^]^ Rho GTPases exist in either an inactive GDP‐bound state or an active GTP‐bound state, in which the GTPase can interact with downstream factors. Members of Rho‐family GTPases RhoA, Rac, and Cdc42 are known to regulate the assembly of the actin structures required for cell motility in glioma cells, which are stress fibers, lamellipodia, and filopodia.^[^ ^95^ ^]^ Rho is mainly involved in the formation of stress fibers and focal adhesions, Rac activates the formation of lamellipodia and Cdc42 is involved in the formation of filopodia and is also known to activate Rac.^[^ ^96^ ^]^ Rho GTPases promote myosin‐actin interactions through Rho‐associated coiled‐coil kinase (ROCK). Direct phosphorylation of myosin light chain (MLC) and myosin light chain phosphatase (MLCP) by ROCK leads to an increase on the level of phosphorylated myosin light chain, which contributes to contractility.^[^ ^97^ ^]^

#### 7.2.2 Angiogenesis

Angiogenesis is a key event in the progression of malignant gliomas.^[^ ^98^ ^]^ For the diagnosis of GBM, microvascular proliferation, and necrosis are required.^[^ ^7^ ^]^ GBM, which displays high endothelial cell hyperplasia and vascular proliferation, has been reported to be the most angiogenic brain tumor.^[^ ^99^ ^]^ Microvessel density is an indirect measure of angiogenesis and correlates with patient prognosis in astroglial brain tumors, with GBM being the one with the highest microvessel density and the poorest survival outcome.^[^ ^100^ ^]^ The aberrant vascularity of these tumors, consisting of excessive and disorganized blood vessels, allows glioma cells to meet their metabolic requirements (oxygen, nutrient uptake, and waste disposal) and the creation of an abnormal vascular stem cell niche that maintains the cancer stem cells.^[^ ^32^ ^]^ Tumor invasion probably precedes sprouting neoangiogenesis and might be associated with the cooption of pre‐existent blood vessels, which has been proposed as a potential explanation of the limited effect of antiangiogenic treatment.^[^ ^101^ ^]^ New blood vessels formation in brain tumors occurs through different mechanisms besides angiogenesis, such as vascular cooption, vasculogenesis by recruitment of endothelial precursors of bone marrow, vascular mimicry and GSC endothelial differentiation.^[^ ^102^, ^103^, ^104^ ^]^

Blood vessels formation through angiogenesis is regulated by a balance between proangiogenic and antiangiogenic molecules that mediate the angiogenic switch.^[^ ^105^ ^]^ Vascular endothelial growth factor (VEGF) is the main factor orchestrating glioma angiogenesis. When quiescent vessels sense the angiogenic signal of VEGF, pericytes detach from the vessel wall and liberate themselves from the basement membrane through the action of MMPs. VEGF increases vascular permeability, leading to extravasation of plasma proteins and deposition of proangiogenic matrix proteins. Endothelial cells migrate onto this matrix in response to VEGF and other proangiogenic cytokines and assemble themselves to eventually form mature vessels. Hypoxia is the most potent activator of angiogenic mechanisms in brain tumors, as it is a potent stimulator of HIF‐1, which enhances VEGF expression through a HIF‐1α binding site in VEGF promoter.^[^ ^106^ ^]^ There are other factors besides VEGF that stimulate angiogenesis in GBM, such as PDGF, FGF, angiopoietin‐1, angiopoietin‐2 (Ang‐2), DLL4, integrins, IL‐8, and SDF1.^[^ ^102^ ^]^ Proangiogenic mediators are opposed by the action of antiangiogenic factors, such as endostatin, tumstatin, thombospondins, angiostatin, interferons, and tissue inhibitors of metalloproteinases.^[^ ^107^ ^]^ When stimulatory factors outweigh inhibitory factors, the angiogenic switch turns on and leads to vessel formation.

Normal brain vasculature is composed of endothelial cells, pericytes and astrocytes. These cells maintain a unique structure in the brain parenchyma: the BBB, which selectively restricts the exchange of molecules between the intracerebral and extracerebral circulatory systems. Rapid proliferation of brain tumors within the brain parenchyma compromises BBB structure and function. This causes the accumulation of fluid and plasma proteins peritumorally and in the surrounding brain, which is a confined space lacking the lymphatic vasculature needed for draining excess fluid. The fluid leakage leads to interstitial hypertension within the tumor and accumulation of fluid outside the tumor, resulting in vasogenic brain edema, which is a major cause of morbidity in GBM patients.^[^ ^102^ ^]^ New blood vessels formed in brain tumors acquire morphological abnormalities that constitute diagnostic features, especially for GBM, whose microvasculature appears as “glomeruloid bodies.” These resemble renal glomeruli and are tufted aggregates of newly sprouted vessels lined by highly proliferative endothelial cells and surrounded by basal lamina and an incomplete layer of pericytes.^[^ ^108^ ^]^

**Figure 5:**
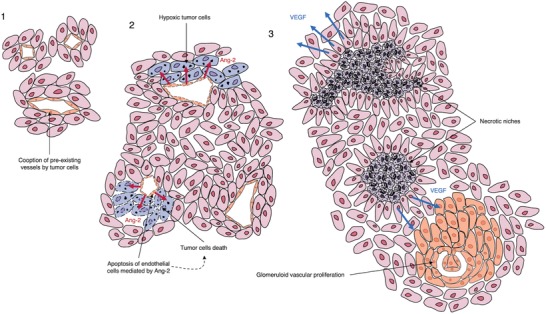
Possible mechanism for glomeruloid bodies formation. 1) Low‐grade infiltrating glioma cells coopt pre‐existing microvessels. 2) As the tumor grows, endothelial cells try to resist cooption by releasing Ang‐2, which leads to apoptosis of these cells in the absence of VEGF. Apoptosis of endothelial cells then causes tumor cells to become hypoxic and eventually necrotic, forming initial foci of necrosis. 3) Necrotic niches become surrounded by tumor cells, forming the pattern of pseudopalisading necrosis. Pseudopalisade tumor cells upregulate the expression and secretion of VEGF, which acts on nearby endothelial cells to promote vascular proliferation, leading to the formation of glomeruloid structures.

When WHO grade III astrocytomas progress to WHO grade IV GBM, two major changes occur in tumor biology: 1) necrosis appears as the result of extensive hypoxia in tumor tissue and 2) microvascular hyperplasia emerges as the hypoxia‐induced angiogenic response.^[^ ^109^ ^]^ Rapid proliferation of tumor cells causes the center of the tumor to become hypoxic and necrotic. Vaso‐occlusive events probably contribute to hypoxia and necrosis too. In fact, it was observed that cooption of pre‐existing vasculature by tumor cells leads to upregulation of angiopoietin‐2 (Ang‐2) expression in co‐opted endothelial cells, which causes apoptosis in endothelial cells, vascular regression, and collapse in the absence of VEGF.^[^ ^110^ ^]^ Tumor cells in immediate proximity of degenerated vessels begin to die, forming initial foci of necrosis. These foci become surrounded by tumor cells which eventually form pseudopalisade and upregulate the expression of VEGF, leading to vascular hyperplasia, including glomeruloid vascular proliferation.^[^ ^108^ ^]^ In contrast to this sequence of vessel cooption, vessel regression and angiogenesis, real‐time data suggest a dynamic interplay of vessel cooption, and angiogenesis in tumor evolution. Invasive glioma cells remodel pre‐existing microvessels at the site of contact.^[^ ^111^ ^]^ Pre‐existing capillaries split in two at the site of physical interaction between glioma cells and blood vessels, which complies with intussusceptive growth.^[^ ^112^ ^]^ Furthermore, tumor cells are able to pull adjacent vessels into the tumor nodules, looping and coiling them up, thus leading to the formation of a chaotic and tortuous intratumoral vessel network that resembles renal glomeruli appearance (**Figure** **5**).^[^ ^113^ ^]^ Kinetics of capillary loops and glomeruloid bodies formation in vivo supports this hypothesis. Thus, invading glioma cells are capable of remodeling pre‐existing vasculature in different ways in order to achieve tumor growth and dissemination. This reciprocal interplay of brain microvessels and invasive glioma cells would explain the interdependence of glioma angiogenesis and invasion, besides previous substantial evidence of the linkage between these two events.^[^ ^111^ ^]^ Whether glomeruloid bodies represent a more aggressive, dysfunctional or abortive form of angiogenesis still remains an open question, and further studies are needed to determine biological importance of these structures.

## 8 Clinical Management of Glioblastoma: New Diagnosis Opportunities and Therapeutic Strategies

The fact that GBM showcases high tumor heterogeneity and the ability to cross the BBB makes it difficult to treat and causes current therapies to be very aggressive and ineffective. These two factors affect treatment response, leading to the acquisition of resistance in GBM patients. Recent advances regarding new‐generation sequencing (NGS) techniques have given rise to the identification of specific molecular features of GBM that allow a better understanding of the molecular pathogenesis of the disease. Integration of information regarding exosome sequences, DNA copy number, epigenetics, mRNA, miRNA, and protein expression has led to the identification of prognosis subgroups of diffuse gliomas in a more precise way than histology had done.^[^ ^114^ ^]^ As a consequence, multiple diagnostic, prognostic and predictive biomarkers have been suggested. Some biomarkers are still being evaluated, while other ones are commonly used in clinical tests in order to identify GBM patients: O^6^‐methylguanine methyltransferase (MGMT), IDH, epidermal growth factor receptor (EGFR), VEGF, p53, PTEN, p16INK4a gene, phospholipid metabolites, cancer stem cells, and imaging biomarkers.^[^ ^115^ ^]^

### 8.1 miRNA as Diagnostic Biomarkers in Glioblastoma: Liquid Biopsy

Although the Verhaak et al.'s classification of GBM is widely used and accepted,^[^ ^6^ ^]^ recent studies show evidence that a more accurate classification based on miRNA profiling is possible. miRNAs are involved in essential pathways in GBM (proliferation, differentiation, apoptosis, migration, and angiogenesis) and have been associated with patient survival and therapy response. This is why, in the last few years, there has been growing interest in their use as biomarkers, due to their relevance in the genetic network that directs the oncogenesis of GBM. Thus, 15 miRNAs with altered expression in GBM have been identified as potential biomarkers: miR‐21, miR‐221, miR‐15‐a/b, miR‐182, miR128‐a/b, miR‐20a, miR‐125b, miR‐106‐a/b, miR‐17, miR‐27a, miR‐99‐a/b, miR‐130‐a/b, miR‐25, miR‐23a, and miR‐10b.^[^ ^116^ ^]^

In the human body, many different cell types, including tumor cells, can communicate with other cells through chemical and electrical signals. But cellular communication is not reduced to these “classical” and widely known systems; since 1983, a new form of cellular communication based on the circulation of nanoparticles has been known. These particles, known as exosomes, contain a cargo from within the cell and leave the cell by exocytosis.^[^ ^117^ ^]^ The cargo includes proteins and nucleic acids as a footprint of its cellular origin, shows specific integrins expression patterns that direct the particles to concrete target cells and are secreted actively by mammalian cells, especially, cancer cells.^[^ ^118^ ^]^

In GBM, exosomes have been discovered recently: in 2008, Skog et al. demonstrated that the microvesicles released by GBM tumor cells contained mRNA, microRNA and angiogenic proteins. These microvesicles are incorporated by normal cells, which can translate the mRNA from within, in such a way that the exosomes constitute a mechanism of propagation of the genetic material of the tumor in the microenvironment. In addition, exosomes can be found in the serum of GBM patients, so a blood test would provide information about mutations and splicing variants of mRNA and microRNA that are characteristic of tumor formation and progression, and their quantification would serve as a tool for monitoring response to therapies.^[^ ^119^ ^]^ This technique is known as liquid biopsy and is a noninvasive method for detecting tumor biomarkers. In spite of how promising it seemed and its usefulness for the detection of some cancers, in the case of brain tumors, its progress has been very limited, since the levels of circulating biomarkers are not very high, due to the obstacle posed by the BBB. A recent study showed that the use of focused ultrasound in murine GBM could increase the release of biomarkers from the tumor mass in the CNS into the bloodstream. This technique, in addition to being noninvasive, allows the selective release of biomarkers from specific areas of the tumor, which may help in understanding the spatial heterogeneity of GBM.^[^ ^120^ ^]^

### 8.2 Available and Promising Therapeutic Strategies for Glioblastoma

Despite advances in the knowledge of molecular biology of GBM and genomics, this progress has not been transferred to the clinic in the same way as with other tumors. The great tumor heterogeneity of GBM is the main obstacle in this path, as it makes the effectiveness of existing and developing therapies very difficult and ends up resulting in the acquisition of resistance to these therapies. This heterogeneity manifests itself in intratumoral, intertumoral and spatial form, so it could be said that each GBM is a different disease that requires personalized treatment and follow‐up. This is, of course, the utopia pursued by all current medical research, but the road ahead is still long, and current treatment options are limited. Conventional treatment for GBM consists of surgery for complete resection followed by radiation therapy and adjuvant chemotherapy with TMZ, which can be combined with alternating electric fields of intermediate frequency. If recurrence occurs, treatment options are more limited and include nitrosoureas and bevacizumab, although the latter is not approved for use in Europe.^[^ ^121^ ^]^ Although progress in the search for new therapeutic strategies is limited, numerous targets and clinical trials are still being proposed to shed light on this devastating disease. **Table** **3** summarizes several ongoing clinical trials, some of which will be discussed below.

**Table 3:** Ongoing clinical trials incorporating experimental drugs for GBM treatment

ClinicalTrials.gov ID (other ID)	Experimental treatment	Condition	Control or comparator treatment	Sponsor	Patients, *n*	Study Phase	Primary outcome measures
Drugs targeting growth factor receptors							
NCT02573324 (Intellance 1)	ABT‐414, RT and TMZ	Newly diagnosed	Placebo, RT and TMZ	Abbvie	640	Phase II/III	OS
NCT02343406 (INTELLANCE 2)	ABT‐414 alone or ABT‐414 + TMZ	Recurrent	Lomustine alone or TMZ alone	Abbvie	260	Phase II	Cmax, PFS, OS, AUC and others
NCT03296696	AMG 596	Recurrent, newly diagnosed	–	Amgen	82	Phase I	*N* subject with adverse events
NCT03618667	GC1118	Recurrent	–	Samsung Medical Center	23	Phase II	PFS6
NCT03603379	C225‐ILs‐dox	Recurrent	–	University Hospital, Basel, Switzerland	9	Phase I	Ratio of C225‐ILs‐dox concentration
NCT03231501	HMPL‐813 (epitinib)	NA	–	Hutchison Medipharma Limited	29	Phase I	ORR
NCT03631836 (MARELLE01)	GS5745	Recurrent	–	Assistance Publique Hopitaux De Marseille	34	Phase I	DLT
NCT01903330	ERC1671/GM‐CSF/Cyclophosphamide + bevacizumab	Recurrent	Placebo injection/placebo pill + bevacizumab	Daniela A. Bota	84	Phase II	Safety
NCT03722342	TTAC‐0001 and pembrolizumab	Recurrent	–	PharmAbcine	20	Phase I	DLT, AE, ADA
NCT03856099	TTAC‐0001	Recurrent			36	Phase II	AE
Drugs targeting DNA repair and cell cycle control pathways							
NCT03107780	AMG‐232	Recurrent, newly diagnosed	–	National Cancer Institute	86	Phase I	PK, MTD
NCT02345824	LEE011 (ribociclib)	Recurrent	–	University of Virginia	3	Phase I	Inhibition of CDK4/CDK6 signaling pathway in cell proliferation
NCT02255461	PD‐0332991 (palbociclib isethionate)	Recurrent	–	Pediatric Brain Tumor Consortium	35	Phase I	MTD, AE
NCT03581292	ABT‐888 (veliparib), RT and TMZ	Newly diagnosed	–	National Cancer Institute	115	Phase II	EFS
NCT02152982	TMZ and veliparib	Newly diagnosed	TMZ and placebo	National Cancer Institute	440	Phase II/III	OS
NCT01514201	Veliparib, TMZ, 3D‐CRT, IMRT	Newly diagnosed	–	National Cancer Institute	66	Phase I/II	MTD, feasibility, OS
NCT03233204	Olaparib	NA		National Cancer Institute	49	Phase II	ORR
NCT01390571	Olaparib + TMZ	Recurrent	–	Cancer Research UK	34	Phase I	Detection of olaparib in tumor tissue, MTD, toxicity profile, DLT
PARADIGM‐2	Olaparib + RT + TMZ (methylated MGMT) or olaparib + RT (unmethylated MGMT)	Newly diagnosed	–	Cancer Research UK	68	Phase I	
NCT03212742	Olaparib + TMZ+ IMRT	NA	–	Centre Francois Baclesse	79	Phase I/II	RP2D
NCT02974621	Olaparib + cediranib maleate	Recurrent	Bevacizumab	National Cancer Institute	70	Phase II	PFS6
Drugs targeting epigenetics and tumor metabolism							
NCT02073994	AG‐120 (veliparib)	NA	–	Agios Pharmaceuticals, Inc.	170	Phase I	AE, MTD, RP2D
NCT02481154	AG‐881 (vorasidenib)	NA	–	Agios Pharmaceuticals, Inc.	150	Phase I	AE, MTD, RP2D
NCT02273739	AG‐221 (enasidenib)	NA	–	Celgene	21	Phase I/II	AE, MTD, RP2D
NCT02381886	IDH305	NA	–	Novartis Pharmaceuticals	166	Phase I	DLT
NCT03030066	DS‐1001b	NA	–	Daiichi Sankyo Co., Ltd.	60	Not Applicable	% of patients with DLT
NCT02746081	BAY1436032	NA	–	Bayer	81	Phase I	AE, MTD, RP2D
NCT02454634	IDH peptide vaccine			National Center for Tumor Diseases, Heidelberg	39	Phase I	Safety, tolerability, immunogenicity
NCT03426891	Pembrolizumab + vorinostat + TMZ + RT	Newly diagnosed	–	H. Lee Moffitt Cancer Center and Research Institute	32	Phase I	MTD
NCT00731731	RT + vorinostat + TMZ	Newly diagnosed	–	National Cancer Institute	125	Phase I/II	MTD, OS
NCT00268385	Vorinostat + TMZ	NA	–	National Cancer Institute	83	Phase I	MTD
NCT00555399	Vorinostat + isotretinoin/TMZ+ isotretinoin/vorinostat +isotretinoin + TMZ	Recurrent	–	M.D. Anderson Cancer Center	135	Phase I/II	MTD
Drugs targeting angiogenesis							
NCT01290939	Lomustine + bevacizumab	Recurrent	Lomustine	European Organisation for Research and Treatment of Cancer	433	Phase III	OS
NCT03025893 (STELLAR)	Sunutinib	Recurrent	Lomustine	VU University Medical Center	100	Phase II/III	PFS6
NCT01931098	Topotecan + pazopanib	Recurrent	–	National Cancer Institute	35	Phase II	PFS6, PFS3
Immunotherapies							
NCT02078648	SL‐701 + poly‐ICLC + bevacizumab	Recurrent	–	Stemline Therapeutics, Inc.	74	Phase I/II	Safety, tolerability, OS12, ORR
NCT02844062	Anti‐EGFRvIII CAR T cells	Recurrent	–	Beijing Sanbo Brain Hospital	20	Phase I	Safety
NCT02649582 (ADDIT‐GLIO)	Dendritic cell vaccine + TMZ	NA	–	University Hospital, Antwerp	20	Phase I/II	OS
NCT02798406	DNX‐2401 + pembrolizumab	NA	–	DNAtrix, Inc.	49	Phase II	ORR
NCT03043391	Polio/Rhinovirus Recombinant (PVSRIPO)	Recurrent	–	Istari Oncology, Inc.	12	Phase I	Percentage of participants with unacceptable toxicity
NCT02414165	Toca 511/Toca FC	Recurrent	Lomustine, TMZ or Bevacizumab	Tocagen Inc.	403	Phase II/III	OS
NCT02550249 (Neo‐nivo)	Nivolumab	Newly diagnosed and recurrent	Nivolumab	Clínica Universidad de Navarra	29	Phase II	Expression of PDL‐1
NCT02336165	MEDI4736 alone or MEDI4736 + bevacizumab or MEDI4736 + RT	Newly diagnosed and recurrent	–	Ludwig Institute for Cancer Research	159	Phase II	OS, PFS6
NCT03174197	Atezolizumab + TMZ or atezolizumab + TMZ + RT	Newly diagnosed	–	M.D. Anderson Cancer Center	60	Phase I/II	DLT, OS, AE

ADA: anti‐drug antibody; AE: adverse events; AUC: area under the curve; DLT: dose limiting toxicity; EFS: event‐free survival; IMRT: intensity modulated radiation therapy; MTD: maximum tolerated dose; NA: not available; ORR: objective response rate; OS: overall survival; OS12: overall survival at 12 months; PFS: progression‐free survival; PFS3: progression‐free survival at 3 months; PFS6: progression‐free survival at 6 months; PK: pharmacokinetics; poly‐ICLC: polyinosinic–polycytidylic acid stabilized with polylysine and carboxymethyl cellulose; RP2D: recommended phase II dose; RT: radiation therapy

#### 8.2.1 Targeting Growth Factor Receptors and Their Downstream Signaling Pathways

These are molecules that inhibit overactivated signaling pathways that allow tumor formation and progression, including targets such as EGFR or PDGFR. Aberrations in EGFR are present in ≈50% of GBMs. Rindopepimut is an EGFRvIII peptide vaccine that showed promising results in preclinical models but did not succeed in transferring to human clinic, with final analysis showing no increase in OS in ACT IV Phase III trial.^[^ ^122^ ^]^ ABT‐414 is an antibody‐drug conjugate (ADC) consisting of an anti‐EGFR monoclonal antibody conjugated to the tubulin inhibitor monomethylauristatin. ABT‐414 showed significant therapeutic benefit in GBM patient derived xenografts in combination with standard‐of‐care TMZ and radiation and advanced to phase I/II clinical trials.^[^ ^123^ ^]^ Progression free survival (PFS) in phase I study was 30.8% and clinical development of ABT‐414 is ongoing in randomized phase II trial for recurrent GBM (NCT02343406) and randomized phase IIb/III trial for newly diagnosed GBM (NCT02573324).^[^ ^124^ ^]^ *PDGFRA* amplification is found in nearly 15% of GBMs. Dasatinib is a multikinase inhibitor targeting PDGFR, among other kinases. It was found to be ineffective in patients with recurrent GBM.^[^ ^125^ ^]^ Trials evaluating other multikinase inhibitors did not show significant clinical benefit in GBM.^[^ ^126^ ^]^

#### 8.2.2 Targeting DNA Repair and Cell Cycle Control Pathways

Disruption of p53 and RB is present in more than 80% of GBM. In addition to mutation or deletion, inactivation of p53 may be due to amplification of *MDM2* or *MDM4*. Thus, inhibition of MDM2 has been proposed as a possible strategy to restore p53 function. AMG‐232 is a MDM2 inhibitor that is being evaluated in phase I clinical trial (NCT03107780). Cell cycle disturbances are due, among other factors, to the inactivation of *CDKN2A/CDKN2B* and *RB1*, as well as to the amplification of *CDK4* and *CDK6*.^[^ ^30^ ^]^ Clinical trials evaluating CDK4/6 inhibitors are ongoing: ribociclib (NCT02345824) and palbociclib (NCT02255461) are two of these molecules. Although the latter has been shown to be ineffective for recurrent GBM, patients had been heavily pretreated, and authors say targeting CDK4/6 pathway may still deserve further exploration.^[^ ^127^ ^]^ Poly (ADP ribose) polymerase (PARP) family of enzymes has a pleiotropic role in DNA repair and has emerged as an attractive target for sensitization of GBM cells to TMZ.^[^ ^128^ ^]^ Clinical trials are ongoing to prove efficacy of PARP inhibitors in treating glioma/GBM patients, alone or in combination with TMZ/radiation therapy. Some of these molecules are veliparib (NCT03581292, NCT02152982, NCT01514201), olaparib (NCT03233204, NCT01390571, PARADIGM‐2, NCT03212742), and pamiparib (NCT03150862, NCT03333915, NCT02361723). WEE1 is a serine/threonine kinase that acts as a gatekeeper against mitotic catastrophe in GBM. Inhibition of WEE1 causes sensitization of GBM cells to DNA damaging agents, including ionizing radiation.^[^ ^129^ ^]^ Combination of the WEE1 inhibitor adavosertib with radiation therapy and TMZ is currently being evaluated (NCT01849146).

#### 8.2.3 Targeting Epigenetics and Tumor Metabolism

*IDH1/2* mutations result in a gain of function, resulting in the production of 2HG, an oncometabolite that interferes with cell metabolism and epigenetic regulation. In addition, these mutations result in a hyper‐methylated phenotype, which can alter chromosome topology and gene expression.^[^ ^49^ ^]^ Mutations alter the catalytic function of enzymes, so they are potential drug targets. Small inhibitory molecules of IDH mutants are being evaluated in clinical trials: ivosidenib (NCT02073994), vorasidenib (NCT02481154), enasidenib (NCT02273739), IDH305 (NCT02381886), DS‐1001b (NCT03030066), and BAY1436032 (NCT02746081). A peptide vaccine for IDH1 is also being evaluated (NCT02454634). Histone deacetylase inhibitors (HDACi) lead to glioma cell death through mitotic catastrophe‐induced apoptosis,^[^ ^130^ ^]^ thus representing and emerging type of therapeutics. Vorinostat is a HDACi that is being evaluated in clinical trials alone or in combination with other drugs/radiation (NCT03426891, NCT00731731, NCT00268385, NCT00555399), despite showing no significant improvement in 6‐month PFS and OS in combination with bevacizumab and TMZ.^[^ ^131^ ^]^ Recently, Meng et al. demonstrated that HDACi panobinostat combined with bromodomain inhibitor JQ1 or OTX015 had synergistical efficacy against GBM cells.^[^ ^132^ ^]^ HDACi givinostat has shown promising results in in vitro and in vivo models. Givinostat counteracts GBM oncophenotype by inducing cell cycle arrest, apoptosis, autophagy‐related nonapoptotic cell death and differentiation, and reduction on GBM stemness potential. In vivo experiments showed that givinostat efficiently passes the BBB and impairs GBM growth in orthotopic xenotransplanted mice.^[^ ^133^ ^]^

#### 8.2.4 Targeting Angiogenesis

Therapeutic manipulation of VEGF/VEGFR is the most studied clinical route in GBM; however, there is currently no effective therapy to stop angiogenesis in GBM, due to the complexity of the angiogenic process in this tumor. Failure of antiangiogenic strategies to improve OS, both in patients diagnosed for the first time and in cases of recurrence, demonstrates that vascular pruning is insufficient to stop angiogenesis in GBM.^[^ ^134^ ^]^ Bevacizumab is an IgG1 monoclonal antibody directed against VEGF‐A that was approved by the FDA as monotherapy for recurrent GBM in 2009, but the European Medicines Agency declined approval due to lack of a bevacizumab‐free control arm in clinical trials.^[^ ^121^ ^]^ Several studies showed later that bevacizumab does not improve overall survival in newly diagnosed GBM patients.^[^ ^135^, ^136^ ^]^ Bevacizumab use is restricted to recurrent GBM patients, although they tend to relapse during treatment.^[^ ^137^ ^]^ A common strategy to treat recurrent GBM is the combination of bevacizumab with a cytotoxic drug. This strategy indicated potential survival benefit for classical subtype GBMs patients when treated with bevacizumab and lomustine (a nitrosourea) in a phase II clinical trial.^[^ ^138^ ^]^ Other anti‐angiogenic agents have been proposed for GBM treatment, such as VEGF receptor tyrosine kinase inhibitors, PDGF receptor tyrosine kinases inhibitors, protein kinase C inhibitors, MMP inhibitors and proteasome inhibitors. Unfortunately, none of them has demonstrated enough effectiveness in phase I and II clinical trials.^[^ ^139^ ^]^ Progress in the development of anti‐angiogenic strategies in GBM is far from that achieved in other types of tumor, and rapid acquisition of resistance is one of the main obstacles. Vessel cooption is thought to be involved in resistance of GBM cells to anti‐angiogenic treatments, including anti‐VEGF treatment. In a recent study, Voutouri et al. constructed and validated a mathematical model that reveals dynamics of tumor vessel cooption and predicts that low or high VEGF blockade does not lead to vessel pruning, which is consistent with the hypothesis that vessel cooption can act as a mechanism of resistance to anti‐angiogenic treatment in GBM. The study also provides guidelines for effective therapeutic strategies combining inhibition of both angiogenesis and cooption.^[^ ^140^ ^]^ A better understanding of this and other mechanisms of resistance to anti‐angiogenic therapies could help in the search of new targets and drugs that would involve alternative pathways to stop tumor vascularization and improve long‐lasting response, which is the greatest challenge of anti‐angiogenesis in GBM.

#### 8.2.5 Immunotherapies

Apart from Rindopepimut, clinical trials are underway with other vaccines that do not require individualization for each patient. Tumor antigens can be combined in multipeptide vaccines, reducing the possibility of antigen‐negative cells remaining.^[^ ^141^ ^]^ SL‐701 is a multipeptide vaccine directed against IL‐13Ra2, survivine and Epha2, and currently undergoing phase I and II trials (NCT02078648). Other immunotherapies require patient's tissue for the generation of the vaccine, as it is the case with autologous dendritic cell therapy. Results are not perfectly concordant, but several studies show significant benefit in OS and PFS.^[^ ^142^ ^]^ Immune checkpoint inhibitors are negative immunological regulators. Cytotoxic T lymphocyte‐associated antigen 4 (CTLA‐4) and programmed cell death protein 1 (PD‐1) are the most studied immune checkpoint receptors. A few immune checkpoint inhibitors have been approved by the FDA for their use in several types of cancer.^[^ ^143^ ^]^ This is the case of ipilimumab and tremelimumab (CTLA‐4 inhibitors); nivolumab and pembrolizumab (PD‐1 inhibitors); atezolizumab, avelumab; and durvalumab (PD‐L1 inhibitors).^[^ ^144^ ^]^ Chimeric antigen receptor T (CAR‐T) cell therapy is an emerging immunotherapeutic strategy in oncology. Four antigens have been pursued in CAR clinical trials of GBM: EGFRvIII, human epidermal growth factor receptor 2 (HER2), interleukin receptor 13Rα2 (IL‐13Rα2) and erythropoietin‐producing hepatocellular carcinoma A2 (EphA2).^[^ ^145^ ^]^ Finally, viral therapy is also recognized as a form of immunotherapy. Oncolytic viruses can selectively kill cancer cells without causing damage to normal tissues. The most promising viruses undergoing clinical trials currently are DNX‐2401 (NCT02798406), PVS‐RIPO (NCT03043391), and Toca 511 (NCT02414165).^[^ ^146^ ^]^

### 8.3 Nanomedicine in the Treatment of Glioblastoma

The reason why conventional chemotherapy treatments for brain tumors fail to avoid remission of the tumor after surgical resection followed by chemotherapy is that penetration and retention of the drug in brain tissue is poorly achieved. However, blood vessels in solid tumor show some weaknesses that can be exploited in order to enhance drug delivery to the tumor. These are extensive angiogenesis, extensive extravasation, defective vascular architecture and impaired lymphatic clearance from the interstitial space of tumor tissues. They all enhance the permeability of blood vessels in tumor tissues and contribute to retention of macromolecules and other particles. Maeda coined the term enhanced permeability and retention (EPR) to describe this effect.^[^ ^147^ ^]^ Thanks to EPR effect, some nanomaterials have been shown to be able to cross the BBB and be retained in brain tumor tissue. Nanoparticle‐mediated delivery systems allow for a controlled local release within brain tissue. Computational models can help to understand how physicochemical properties of nanoparticles affect therapeutic delivery and efficacy, thus allowing to optimize the design of nanoparticles for the treatment of brain tumors.^[^ ^148^ ^]^

Regarding GBM, different approaches have been explored in the past few years. Nonenergy dependent pathways (passive uptake) and energy‐dependent pathways (active uptake through convection‐enhanced delivery, CED) have been considered for drug delivery, in a mission to identify less invasive and safer ways to apply this therapeutic strategy. Also, different delivery strategies have been developed, as viral delivery vehicles, nonviral delivery vehicles and multifunctional delivery systems.^[^ ^149^, ^150^ ^]^ Although nanoparticles are able to cross the BBB, active targeting to tumor tissue still needs to be improved. Polymeric nanoparticles and CED, polymeric micelles and CED, and liposomal nanoparticles and active tumor targeting are plausible therapeutic strategies.^[^ ^151^ ^]^

Recent advances have been made in nano‐mediated delivery of double‐stranded RNA (dsRNA) (gene therapy), antiangiogenic factors and immunoconjugates. Magnetic nanoparticles show advantages over other materials that have made them a suitable material for nanomedicine. They are easy and cheap to produce, are physically and chemically stable and can be employed as contrast agents in magnetic resonance imaging (MRI). Grabowska et al. recently reported the successful use of magnetic nanoparticles in delivering dsRNA coated with polyethyleneimine for RNA interference therapy of GBM on human GBM U‐118 MG cell line. These features should make magnetic nanoparticles to be considered as a robust theragnostic platform.^[^ ^152^ ^]^ Antiangiogenic factors face challenges to their use in GBM treatment, mostly due to their inability to cross BBB, thus not reaching therapeutic concentrations in the brain. High doses of antiangiogenic factors lead to systemic toxicity, making it difficult to develop effective therapeutic strategies. Clavreul et al. (2019) discuss four local or systemic nonviral methods for antiangiogenic factor delivery, namely CED devices, implantable polymer devices, nanocarriers and cellular vehicles.^[^ ^153^ ^]^ Even though they have all been evaluated in orthotopic GBM models, only CED devices have reached clinical practice. Treatment with immune checkpoint inhibitor antibodies against CTLA‐4 and PD‐1 has not reached success due, again, to their inability to cross the BBB. Galstyan et al. carried out in 2019 the first study using polymer‐based nanocarriers with covalently attached immunotherapeutic agents as a GBM treatment through activation of both systemic and local immune response. They successfully delivered a nanoscale immunoconjugate consisting of drug carrier poly(β‐l‐malic acid) covalently conjugated to CTLA‐4 and PD‐1 antibodies to brain tumor cells, which resulted in immune system activation and prolonged survival of intracranial GBM GL261‐bearing mice.^[^ ^154^ ^]^

### 8.4 Integrating Omics Data and Clinical Management of Glioblastoma

In the past few years, high‐throughput technologies (HTT) as microarrays and NGS have become widely developed, accessible and used by groups all around the world to obtain information about cancer genomes, genome profiling, gene expression, tumor initiating cells and a large list of possibilities. The amount of available data coming from HTT has increased massively due to the extended use of these technologies, but managing these data is a great challenge. Many databases contain information and data regarding GBM molecular and genomic features, but the integration of these resources has been poorly developed, which makes it difficult for researches to access and analyze different types of information about GBM. With the attempt to help integration of GBM data and information, Yang et al. have recently developed the open database *GliomaDB*, a web server for integrating glioma omics data and interactive analysis.^[^ ^155^ ^]^ They implemented *GliomaDB* using an open‐source relational database management system called MySQL (https://www.mysql.com), among other tools, which has been widely used for the construction of relational databases and allows for the exchange of information between databases implemented using this system. Although this initiative must be further developed for use optimization, it is a great step toward GBM data integration and management, since it provides access to glioma projects from ten public databases and offers several analytical tools, such as survival analysis, early diagnosis, cluster analysis and co‐expression networks.

As we have previously mentioned across this review, one of the major challenges for GBM treatment is tumor heterogeneity. Multiple aberrations of the same gene occur within the same tumor. Single‐cell (SC) omics are the currently used approach to address this issue. SC omics made the first appearance in GBM research in 2014, when Stieber et al. revealed clonal heterogeneity of GBMs and its implications in glioma progression and treatment response by using SC array‐comparative genomic hybridization,^[^ ^156^ ^]^ and Francis et al. developed a SC sequencing methodology capable of identifying tumor cell subpopulations containing distinct genetic and treatment resistance profiles.^[^ ^157^ ^]^ The latter showed that SC omics can reveal information about phylogenetic relationships among tumor cell subpopulations, thus resolving intratumor clonal evolution at the SC level, which represents a big step toward tackling resistance to GBM treatments.

Recently, Wang et al. identified signaling networks downstream of cancer driver genes in brain tumors by multi‐omics profiling.^[^ ^158^ ^]^ It has been shown that pathways are more relevant than individual genes to cancer progression,^[^ ^159^ ^]^ which makes it worth studying mutations at pathway level. Identification of multi‐omics signatures of GBM has also allowed to establish prognostic subtypes, namely invasive (poor), mitotic (favorable), and intermediate. This new transcriptome‐based classification could lead to a better stratification of patients for therapeutic intervention, enabling rational therapy based on biological phenotype.^[^ ^160^ ^]^

SC and multi‐omics approaches may become key tools for precision medicine in the near future. They allow to characterize single tumors in an integrative way, ranging from the mutations present in different subpopulations to the phylogenetic relationships that lead to the clonal evolution of the tumor that causes resistance to treatments. SC omics have helped deciphering the connection between oncogenesis and CNS development and opens the door to reflection on possible relationships between tumorigenesis and embryonic development.^[^ ^161^ ^]^ The resolution of this technology allows studying processes taking place in tumors with unprecedented accuracy and offers new opportunities to quantitatively address fundamental questions that would otherwise remain unknown. The answers may enable us to face great challenges regarding clinical management of GBM.

## 9 Conclusion

Many efforts have been made to contribute to decipher the molecular biology and genetics underlying the development of GBM. This has led to changes in CNS tumors classification and management of patients, helping to pave the path for personalized therapy. But still, GBM causes the death of thousands of people across the planet every year, with no possibilities to stop the progression of the disease. The understanding of molecular, genetic and metabolic features of GBM is essential for the identification of new potential targets and the development of new therapeutic strategies. But also, the optimization of clinical trials should be an urgent priority in order to properly evaluate thousands of compounds and biological agents that have been developed in the last decade. Many promising therapeutic strategies have shown no clinical benefit when translated to GBM patients. Determining the timing of molecular events leading to GBM progression may be critical for the success of clinical trials. This work gathers and arranges these events as they are known today, but many questions remain and there are pathways yet to be explored. Beyond the features that have been used to classify—and treat—CNS tumors, it may be worth considering new approaches and introducing new elements, such as metabolic alterations. Analyzing the expression of enzymes isoforms and production of metabolites with different cellular functions may help to determine crucial steps in the tumor metabolism and thus targeting them to inhibit tumor growth. In addition to introducing new approaches, it would be appropriate to review the avenues already explored and redirect research to points that may be more productive; this is the case of angiogenesis research, traditionally based on VEGF and now evolving to the search of alternative pro‐ and antiangiogenic pathways. The spatial and temporal tumor heterogeneity of glioblastoma calls for a focus on the interactions that occur in the TME and requires sampling strategies across space and time. The heterogeneity in the niche of the tumor can affect response to therapies. The existing crosstalk between different cellular types and molecular and metabolic pathways suggests that single‐agent therapeutic strategies may result in short term success. New models—like cerebral organoids—addressing both spatial and temporal heterogeneity may allow for the understanding of evolutionary dynamics and provide insight into the molecular mechanisms underlying GBM progression and recurrence. Perhaps the introduction of these new approaches will bring fresh air and allow the scientific community and patients to perceive that research on GBM is moving steadily forward.

## Conflict of Interest

The authors declare no conflict of interest.
